# Supplementary material for: Are carbon emissions trading and green financial instruments synergistic? -Comprehensive quantitative research based on content analysis
Source: PLoS One. 2024 Mar 7;19(3):e0298601. doi: 10.1371/journal.pone.0298601 (PMC10919601; doi:10.1371/journal.pone.0298601)
Supplement: S1 Data — (ZIP) [file pone.0298601.s001.zip › Data/地级市Z_F工作报告.zip!/╜¡╬≈/╥╦┤║╩╨/└┤╫╘╣1⁄2╓┌║┼í╛┬φ┐╦╩2╛▌═°í┐_2004.docx]

各位代表：

现在，我代表市人民政府向大会报告工作，请予审议，并请市政协委员和其他列席会议的同志提出宝贵意见。

一、2003年政府工作回顾

2003年，在市委的正确领导下，全市上下认真贯彻落实党的十六大和十六届三中全会精神以及省委、省政府的决策部署，进一步解放思想，开拓进取，扎实工作，认真破解前进道路上的一系列难题，国民经济快速回升，经济运行质量明显提高，社会事业全面进步，实现了市一届人大五次会议上确定的各项工作目标，主要经济指标均保持两位数增长。完成国内生产总值249.5亿元，增长12.7%，结束了多年低位徘徊的局面，是近8年增幅最高的年份；财政总收入20.6亿元，增长15.5%，是近8年最好水平；全社会固定资产投资80亿元，增长30%。

工业经济高速增长，农业发展势头良好。规模以上工业完成增加值30.9亿元，增长38.2%。三九宜工、丰城电厂、四特集团、丰城矿务局、济民可信、海尔思药业等一批重点企业发挥了重要支撑作用。11个工业园区完成基础设施投入8.93亿元，入园企业565家，上交税金2.47亿元。在工业经济快速增长的带动下，三次产业结构由上年的32.8:34.5:32.7提升到30.2:37.8:32。10大农业主导产业预计实现综合产值23.14亿元，上交税收7000万元，分别增长13.3%和24.5%。农产品基地建设顺利推进，通过省级无公害基地认证39个，通过有机食品认证12个。完成工业原料林基地建设26万亩，中药材种植面积16.8万亩，花卉苗木种植面积6.8万亩。

基础设施建设力度加大，城市化进程进一步加快。全年共投资4.16亿元，改造国省道383.8公里、农村公路783公里。实施了农网改造、农村公路改造、退耕还林、旧村改造、农村沼气等工程项目，完成农网改造投资3.49亿元，顺利实现了城乡电网同网同价，完成退耕还林36.4万亩，151个村完成旧村改造，靖安县被列为全国沼气工程建设示范县。水利基础设施建设力度加大，完成投入2.38亿元，一批重点水利工程除险加固和潦河灌区配套改造进展顺利。城市化进程加快，城市化率达到29%，比上年提高2个百分点。城市经营取得突破，全面推行了国有经营性土地使用权招标、拍卖、挂牌出让制度，全市土地收储7858亩，国有土地出让交易总额7.6亿元，其中市本级土地收储1760亩，实现土地收入3.36亿元，比上年增长71%。宜春中心城建设步伐进一步加快，25项城建重点工程有7项完工或基本完工，完成投资近7亿元，道路改造面积18万平方米，5条主干道的沥青路面完成铺设。开展了“迎农运，创‘三城’”活动，完成39条小街小巷改造，拆除违章建筑面积2.5万平方米。丰城、樟树、上高等县市新城建设力度加大，城市面貌发生了显著变化。

开放型经济势头强劲，各种投资大幅增长。全年实际引进市外资金93.2亿元，增长93%；实际引进境（国）外资金8391万美元，增长86.2%；外贸出口3100万美元，增长41.9%；对外工程承包完成营业额1040万美元，增长73.3%。市外境外投资方向和质量发生了积极变化，工业项目超过了69%，投资3000万元以上的大项目接近30%。对外交往取得历史性突破，与韩国尚州市签署了开展友好往来备忘录。加大了跑项目、争资金工作力度，丰城电厂、洪屏电站等项目得到了省里的积极支持。民间投资大幅增长，个体工商户和私营企业注册资金达62.51亿元，增长61.1%，其中注册资金1000万元以上的私营企业82户，增加33户。金融对地方经济支持力度进一步加大，金融机构各项存款余额265.69亿元，比年初增加37.05亿元，同比多增3.1亿元；各项贷款余额190亿元，比年初增加14.4亿元，同比多增7.6亿元。

农运会筹办扎实有力，各项工作稳步推进。宾招条件得到很大改善，新增床位3380个，改造床位2700个。47家指定接待单位新建改造投入资金4.8亿元。大型文体表演《田野大风颂》的创作已全部完成，现已将任务分解到16个参演单位排练。根据竞赛工作总规程，编制了各项竞赛计划，试制了部分器材，开始了裁判员培训。体育场馆建设和改造正在全面铺开，已完成工程量3450万元，宜春学院田径场已竣工，学院体育馆主体工程已完工，体育中心游泳馆正在安装设备。新闻宣传工作有所突破，在北京成功召开了新闻发布会。筹资工作进展顺利，成功发行了农运会邮政邮资明信片，共筹资350万元。江西润田公司出资300万元认购了唯一指定饮用水和篮球比赛冠名权。由北京中体同方体育科技有限公司承建的电子信息系统工程全面启动。

人民生活水平逐步提高，社会事业稳步发展。城乡居民收入稳步增加，城镇居民人均可支配收入6515元，增加252元；农民人均纯收入2689元，增加107元。居民消费水平稳步提高，社会消费品零售总额114.3亿元，增长9.6%。进一步加强了社会保障体系建设，“两个确保”全部到位，全市5500多名签协议仍留在中心的下岗职工全部领到了基本生活费，总额达1552万元；为5.8万名国有企业离退休人员按时足额发放了离退休费，总额达2.87亿元。支出低保补助金6134万元，城镇居民最低生活保障人数达12.42万人。支出690万元救助农村特困群众11.56万人，支出5000多万元较好地保障了灾民生产生活。就业和再就业工作有效推进，城镇新增就业人数2万人，城镇登记失业率控制在3.6%以内。科技工作成效突出，获准成为全省首批制造业信息化示范市，列入国家科技计划项目10项，其中健心胶囊的开发与创新研究列入国家重大科技专项。高中教育、职业教育和民办教育发展势头良好，高考成绩连续6年二本以上人数总量居全省第一；宜春学院面向全国24个省、自治区、直辖市招生，提前4年完成既定办学目标；市教育园区建设正式启动，宜春中学新校园已开工建设；宜春职业技术学院已正式组建并按新建制招生，长江理工专修学院进入筹建阶段。文艺创作成果丰硕，10余件作品获省级以上奖励，确定并公布了第一批30处市级文物保护单位，启动了袁州古天文台等文物的修复工程。总结防治非典的经验教训，加强了公共卫生建设，制定并开始实施疾病预防控制体系和医疗救治体系建设，建立了突发公共卫生事件的预警、应急机制和公共卫生长效机制。体育事业蓬勃发展，承办了全国、全省10多项体育赛事，江西省首个全国全民健身中心正式落户宜春。市本级和9县（市）广电网与省广电网成功整合。旅游工作稳步发展，靖安三爪仑森林公园评为3A级景区。人才工作有了新的进步，人才结构比例得到改善，具有初级职称以上的专业技术人员总量增长6.8%。人口和计划生育工作整体水平得到提升，上高县跻身全国先进行列。全市计划生育率92.32%，人口出生率14.38‰，人口自然增长率8.02‰。

各项改革进展顺利，安全稳定形势明显好转。圆满完成农村税费配套改革既定任务，乡镇个数减少20.4%，乡镇行政编制精简23.5%，农村中小学调减17.4%，农村中小学公用经费和危房改造资金安排到位。国企改革已近尾声，全市地方国有工业企业完成改制233户，改制面达75.6%，市直企业改革继续推进，宜春造纸厂、江西机床电器厂改制工作全面完成，宜春齿轮厂改制工作进展顺利。农村卫生综合改革取得新进展，樟树市列为全省新型农村合作医疗改革试点县市。市级机构改革进展顺利，政府工作部门和部门管理机构精简10%。完善了市与县、县与乡财政管理体制，市、县、乡财政分配关系进一步理顺。进一步加大了安全生产专项整治和监督检查力度，全市工矿企业发生事故和死亡人数分别下降42.9%和55.2%，其中花炮企业未发生一起死亡事故，水上交通连续16年无死亡事故，未发生重特大火灾事故，杜绝了特大事故和校园死亡事故，生产、交通、消防三大安全是近11年来最平稳的一年。继续深入开展了“严打”整治斗争，各类刑事案件破获率有所提高。加强了对“法轮功”等邪教组织和犯罪分子的防范和打击，加大了信访、纠纷调解力度，有力地维护了社会稳定。

国家安全、人防建设、民兵预备役、精神文明、民族宗教、新闻出版、审计、统计、档案、史志、老龄和妇女儿童等工作都取得了新的成绩。加强了政府廉政建设和机关作风整顿力度，建立了党风廉政监督员制度，首次在全市聘请了20名党风廉政监督员，在64个部门和单位开展了优质规范服务暨政务环境评议评价活动，对破坏发展环境的人和事进行了严肃查处。民主与法制建设进一步加强，政府坚持向人大报告工作，加强与政协的联系，认真接受人大的法律监督和政协的民主监督，人大代表建议、政协委员提案共221件全部办复。

各位代表，过去的一年，是宜春发展进程中很不寻常的一年。我们面临的困难比预料的大，但取得的成绩比预料的好。在突如其来的非典疫情和各种自然灾害面前，全市上下万众一心，众志成城，预防非典取得了“不发生一例原发病人，力争没有一例输入病人、确保不死一个病人”的阶段性重大胜利，自然灾害损失减少到了最低程度。回顾一年的工作，我们深感成绩来之不易。在此，我谨代表市人民政府，向全市人民致以崇高敬意！向所有参与、支持和关心宜春发展的广大工人、农民、知识分子、医务人员、公安干警、驻宜人民解放军、武警官兵、中央和省驻宜单位、各民主党派、工商联、各人民团体、离退休老同志和海内外朋友，表示衷心的感谢！

在努力推进经济社会发展的进程中，我们也清醒看到，前进的道路上还面临许多矛盾和不足，主要是：尽管工业增长很快，但主导产业不强，大企业不多，财政增收的基础脆弱；尽管农业稳步发展，但抗击自然灾害的能力还不强，农民增收的渠道还不够宽；尽管工业化、城市化进程加快，但提供的就业岗位远远满足不了日益增长的就业需求；尽管投入大幅增长，但融资渠道还不多，资金缺口仍然很大；尽管发展环境进一步改善，但发展环境不优仍是制约经济发展的主要因素，等等。对这些矛盾和不足，我们一定高度重视，继续采取有力措施，加以克服和解决。

二、2004年政府工作安排

2004年，是全面贯彻党的十六届三中全会精神，完善社会主义市场经济体制的第一年，也是全面实施“心圈廊”经济发展构想的重要一年，做好今年的政府工作，具有十分重要的意义。

从2004年宏观经济环境和发展趋势分析，仍将是机遇大于挑战。第一，世界经济的复苏，产业结构调整和国际产业转移步伐加快，为我们加速发展提供了广阔的空间；第二，党的十六届三中全会召开，社会主义市场经济体制进一步完善，将不断增强经济发展的内生活力；第三，国家继续坚持扩大内需的方针，实施积极的财政政策和稳健的货币政策，将创造更好的发展条件；第四，宜春劳动力、水、土地和电力等基本生产要素低成本优势，加上沪瑞高速公路的贯通以及国道、省道改造建设，我们将更加有力地承接长江三角洲、珠江三角洲和闽南三角区产业转移；第五，随着“心圈廊”发展构想的实施，县域经济发展有了更加科学的定位，无论是丰樟高经济圈，还是320国道走廊、山区生态经济走廊，发展势头越来越好，初步探索出了一条加快发展的新路；第六，在历届政府和历任领导的努力下，我们迎来了最富活力、最有人气的时期，发展的基础更加牢实，发展的氛围更加浓厚，不仅各类经济主体抵御短期意外冲击的能力有所提高，而且所蓄积的能量将在今后进一步得到释放。特别是今年我们将举办第五届全国农民运动会，将极大推进宜春城市化进程，带动宜春经济的发展，增强宜春在全国的影响。面对大好形势，我们既要把握机遇，更要用好机遇；既要完善思路，更要创新思路；既要注重理性思考，更要注重实际操作，坚持以改革的思路指导工作，用发展的办法解决问题，用发展的成果凝聚民心，因势利导，因情施策，在进中求快，快中求好。

根据中央经济工作会议精神和省委十一届五次全会部署，今年政府工作的总体思路是：以邓小平理论和“三个代表”重要思想为指导，深入贯彻落实党的十六大和十六届三中全会精神，牢牢抓住发展第一要务，坚持以人为本、诚信立市，落实“五个统筹”要求，树立全面、协调、可持续的科学发展观，大力实施大开放主战略和“心圈廊”发展构想，以举办农运会为契机，以改革统揽全局，努力将工业化、城市化、农业产业化“三化”融为一体，整体推进，实现良性互动，进一步提升经济社会发展的质量和水平，加强社会主义精神文明和政治文明建设，依法行政，维护社会稳定，努力实现经济、政治、文化、社会全面进步。全市经济和社会发展的主要目标预期为：国内生产总值增长12%，财政总收入增长11%，城镇居民人均可支配收入增加400元，农民人均纯收入增加100元，全社会固定资产投资增长20%，实际利用外资增长10%，利用市外资金增长10%，外贸出口增长20%，社会消费品零售总额增长10%，城镇登记失业率控制在3.5%以内，人口自然增长率控制在8.5‰以内，城镇化率达到30%。

围绕上述目标，我们将坚持以科学的发展观指导工作，以正确的政绩观落实工作，以全面的市场观推动工作，突出八个重点，推进九大工程，切实办好十项实事。

统筹经济和社会发展，在八个方面实现新的突破。

（一）强化运作，加快进度，全力以赴办好农运会

按照“声势大于绵阳，接待近于绵阳，环境好于绵阳，大型文体表演优于绵阳”的目标要求，进一步强化大局意识，举全市之力，高标准抓好各项筹备工作，确保农运会举办圆满成功。

健全机制，细化责任。根据农运会的要求，结合我市的实际，抓紧制定各项筹备工作实施方案。同时，实行定人、定岗、定责、定时、定点、定位，切实做到每项工作都有人抓，每人肩上的责任都明确，确保不出任何疏漏。

整合资源，突出重点。举全市之力，整合广告资源、文化资源、人力资源。进一步加大资金筹措力度，强化市场运作，全面启动农运会市场开发项目，最大限度调动方方面面的积极性，鼓励更多的社会团体和个人为农运会作贡献，并积极争取上级的支持与帮助。加大宣传造势力度，尽快启动市外、省外宣传，把市区所有广告媒体和宣传载体使用权收归市政府，农运会结束前由筹委会统一安排使用。抓好开幕式大型文体表演，做好各参演单位和参演人员的工作，确保演出成功。加快宾招新建改造和场馆建设，在保证质量的前提下，确保6月底前47家宾招指定接待单位和新建、改造体育场馆全部完工并投入使用。

加强统筹，密切配合。进一步动员全市人民积极参与农运会筹办工作，充分发挥筹委会（组委会）的作用，加强指挥调度。大力加强精神文明建设，广泛开展“当好农运东道主，展示文明新形象”活动，提高市民的文明素质。

（二）调整策略，完善举措，大力推进开放型经济

积极融入区域经济一体化进程，调整策略和方式，内外并举，统分结合，突出重点，注重实效，不断开创开放型经济工作新局面。

努力提高招商引资水平。围绕“心圈廊”发展构想，构筑“板块经济”，加强项目工作，建立全市统一的招商项目库和客商资源库。正确把握国际国内资本流动和产业转移的新趋势、新特点，大力实施产业招商和比较优势资源招商，注重引进电子信息、生物制药等高新技术产业和国内外知名企业，吸引有利于壮大支柱产业的关联配套项目，推进物流、旅游和社会事业招商。加强招商队伍和信息平台建设，加大市直部门招商引资力度，改进优化招商手段，积极推行专业招商、以商招商、代理招商和网上招商。继续采取市县联动的方式，瞄准长珠闽、港澳台和东南亚等重点地区，积极开展招商活动。

积极扩大外贸出口。理顺外贸管理体制，落实鼓励出口的相关政策和措施。加强通关服务平台建设，做好海关办事机构申报工作，支持宜春出入境检验检疫局建设。积极帮助具备条件的生产企业申请自营进出口权，重点扶持投资300万美元以上的外商投资企业和年创汇100万美元以上的出口企业发展，做大做强外贸主体，努力实现特色产业优势向出口竞争优势的转变。积极推动出口企业申请各类认证，抓好质量和品牌建设，提高产品的国际竞争力。

深化“双优”工作。全面开展“优化环境年”活动，进一步做好优质规范服务和政务环境评议评价工作，建立对破坏经济发展环境行为的责任追究机制。规范市经济发展服务中心运行机制，健全综合审批“绿色通道”，全面推行招商引资项目跟踪服务。坚持依法办事、规范执法，严格审批和控制有检查、收费权的部门和单位用人计划，核定经费标准，严格经费开支，杜绝乱检查、乱收费行为。继续整顿和规范市场经济秩序，严肃查处“三乱”行为，严厉打击干扰客商正当经营的不法行为，确保客商人身安全和合法权益不受侵害。加强社会信用体系建设，强化全民信用意识，打造“信用宜春”。

（三）培植支柱，构筑平台，努力实现工业化城市化良性互动

以工业化带动城市化，以城市化促进工业化，逐步走出一条具有宜春特色的新型工业化和城市化道路。

加速培植支柱产业簇群。充分发挥樟树“药都”和袁州“省级医药工业园”两大品牌效应，吸引医药企业聚集，做大做强济民可信、海尔思、仁和、樟树医药集团、康力医药物流中心、博士达、华太药业等企业，力争将生物制药业培植成全市第一大产业。扶持以四特酒为主的酒业生产，依托丰富的无公害农产品、绿色农产品和有机农产品，发展壮大食品产业。通过引进资金、技术和先进的管理经验，整合现有生产能力，加速发展以水泥和建筑陶瓷为重点的建材工业。发挥机械行业的研发优势，支持三九宜工、江特、江西龙工、德力西、华伍、清华泰豪、朝日集团等机械企业做强做大。加快工业原料林基地建设，保证竹木加工业的良性发展。积极支持宜春钽铌矿、丰城电厂、丰城矿务局、江西盐矿等中央、省属企业做大做强。十分重视信息化在工业化中的倍增和催化作用，不失时机地推进信息化进程，采用高新技术改造传统产业，推动工业化向更高水平攀升。

千方百计加快工业园区建设。按照城市新区和工业新区的要求，进一步完善水、电、气、路建设和绿化、美化、亮化工程。鼓励各类投资主体参与园区的成片开发，突出抓好市经济技术开发区的温州工业园和医药工业园、高安新世纪工业城的太嘉高科技工业园和清华泰豪工业园、奉新的台湾工业园和宁波工业园、宜丰陶瓷工业园等一批园中园建设。创新园区管理体制和运行机制，加速推进管理主体和经营主体的分离，逐步强化园区的招商引资、规划建设、项目审批和企业服务的职能，组建园区经营公司，不断提高园区的市场化运作水平。建立重大项目跟踪服务制度，狠抓项目落实，重点抓好罗宾公司、福源碳素、三福钢铁等一批投资上亿元的重大项目。建立和完善园区的考核体系，努力提高单位面积投资强度、销售收入、财政收入、出口创汇水平，最大限度降低开发成本，缩短投资回收期，引导园区走规模和效益同步发展的路子。力争工业园区新增300个投产企业，到位固定资产投资45亿元，实现销售收入60亿元，上交税金3亿元，安排劳动力6万人。

多层次推进城市化进程。围绕建设赣西经济中心城市的目标，加快宜春中心城周边主通道建设，继续深入开展“迎农运、创‘三城’”活动，完善创建机制，落实创建责任，争取获得“全省创建文明城市工作先进城市”称号，争取通过“省级园林城”和“省级卫生城”检查验收，以崭新的城市面貌迎接农运会召开。同时，推进县城和省级重点镇建设，加快城乡一体化进程。实行积极的人口迁移政策，逐步按固定住所为主要依据申报户口，采用准入条件取代进城人口控制指标，增加城市常住人口。完善土地收购储备制度，高度垄断土地一级市场，建立土地有形市场，所有经营性土地一律实行招标、拍卖、挂牌出让，确保土地升值。引入市场机制，逐步将城市供水、供气、污水处理以及市政、园林、环卫等城市公用设施实行市场化运作、产业化经营，实现城市资产效益的最大化。依法规范和严格拆迁行为，及时妥善解决房屋拆迁中的各类矛盾，维护被拆迁群众的合法权益。

（四）深化改革，创新机制，增强经济发展的内生活力

改革是发展的强大动力。按照社会主义市场经济发展要求，加大体制机制创新力度，统筹兼顾，突出重点，不断推进各项改革，为加快宜春发展注入新的活力。

努力促进个体私营经济更快更好发展。大力扶持骨干私营企业加快发展，鼓励增加投入，在资本经营、发行债券、兼并重组等方面给予支持；大力促进民营科技企业加快发展，帮助争取国家科技计划与资金支持，积极促成民营企业与大专院校、科研机构的合作；大力推动私营企业走外向型发展的路子，鼓励有条件的企业“走出去”。继续清理取消各种歧视性限制，全面落实扶持个体私营经济发展的政策措施。

继续抓好企业改革工作。坚持一厂一策，因企制宜，尽快全面完成国有、集体企业的改制任务。加大重点骨干企业的股份制改造力度，逐步建立起比较规范的现代企业制度。已进入辅导期的江特公司，争取早日上市。同时帮助江猕酒业、青龙高科及早进入上市辅导期。加快市直企业改革步伐，尽快完成宜春齿轮厂的破产改制工作，扶持新公司发展壮大。帮助指导已改制企业健全制度，规范管理，扩大规模，提高效益，巩固改制成果。

加快事业单位改革步伐。变单位人为社会人，实行以聘用制为主的公平竞争的用人机制。将经营理念引入城市管理公用事业领域，利用管理合约、租赁、特许经营权转让、出售国有资产、股份合作、BOT等多种方式，推进供水、供气、污水处理、环卫、园林等公共事业改革，提高管理效率和质量。逐步建立和实施以城市道路为载体的道路养护、绿化养护和环卫保洁综合承包制度。启动市直由政府直接经营的宾招单位改革。

继续推进财税金融体制改革。加强公共财政建设，深化预算编制改革，完善综合预算、零基预算，搞好部门预算试点，深化“收支两条线”改革。继续贯彻稳健的货币政策，不断加大信贷投入，优化信贷结构，降低不良资产比例，努力防范金融风险。积极稳妥做好农村信用社改革，为经济发展创造良好的金融环境。

进一步转变政府职能。以《行政许可法》实施为契机，深化行政审批制度改革，规范审批行为，精减审批事项，简化办事程序，提高办事效率。推行政务公开，全面推行首问责任制、服务承诺制和“一站式”服务制，积极推行电子政务，降低行政成本。进一步理顺政府、企业、市场的关系，由企业自主决定的事项，还权于企业；社会可以承担的事项，交给社会中介组织；属于竞争领域的资源配置，由市场机制去调节。

（五）调优结构，增加投入，巩固农业基础地位

统筹城乡经济社会发展，更多地关注农村，关心农民，支持农业，把解决好农业、农村和农民问题作为政府工作的重中之重，努力开创农村工作新局面。

继续推进农业产业化经营。在提高粮食综合生产能力的基础上，继续推进农业结构调整，按照“一乡一品、一县一业”的思路，继续做优做强10大主导产业。积极培育和引进农业产业化龙头企业，重点抓好江麻集团、江猕酒业、江西维尔宝、奉新碧云米业、江西仁和、青龙高科、世威奶业、锦江有机食品公司等国家级、省级和市级龙头企业。巩固扩大优质农产品生产基地规模，增加基地数量，特别是加快有机农产品、绿色食品、无公害食品基地建设，每个县市区选择1－2个地方开展试点。加强绿色食品和有机农产品品牌保护，争取再申报10－15个有机农产品或绿色食品品牌，并积极为部分名优特地方产品申请国家原产地保护。全面启动中草药种植基地实施GAP认证工作。加快农产品质量检测中心的建设,努力提高农产品质量。

进一步增加农业投入。建立政府主导、统筹使用、社会参与的农业投入新模式，引导民间资本、工商资本和外资投向农业。充分发挥各县市区农经信用贷款担保中心和小额农贷的作用，加大对农业的扶持力度。加快水利设施建设，提高农业抵御自然灾害的能力。加快旧村改造步伐，全面完成旧村改造规划编制，在每个乡镇选择1－3个自然村进行改造试点。大力实施农村沼气工程，推进灌区续建配套和节水示范项目建设，加快农村公路建设和电网改造，改善生产生活条件。

认真落实各项农村政策。继续深化农村税费配套改革，推进县乡机构和农村义务教育体制改革，全面实行涉农税费和价格公示制、农村中小学“一费制”、农村报刊订阅限额制，切实减轻农民负担。继续推进以购销市场化为目标的粮食流通体制改革，增加种粮农民收入，确保粮食安全。做好退耕还林工作，及时兑现钱粮补助，加快造林进度，确保完成退耕还林任务。完善农业科技推广体系，加强农经网建设，大力发展农村专业合作组织和行业协会，进一步提高农民组织化程度。加强农资市场监管，依法足额落实被征地农民的征地补偿费,切实保护农民利益。

（六）突出重点，整合资源，大力发展第三产业

增强综合服务功能，促进第三产业健康发展，努力提高三产在国民经济中的比重。

着力发展现代服务业。科学制定与城市定位相适应、与城市总体规划相协调的现代服务业发展规划，优化布局，突出重点，加快发展。以示范小区建设为载体，发展社区服务业。规范发展房地产二级市场，加强和完善物业管理，促进房地产业健康发展。巩固商贸、餐饮等传统服务业，拓展金融、保险、电信服务业领域，增强城市综合功能，积极推进信息技术在各行各业的广泛应用，提升信息服务业的发展水平。

着力建设现代物流中心。规划与整合现有批发市场，对经营项目重复的市场进行适当调整，重点培育发展一批市场前景好、流通规模大、辐射力强、交易方式先进的农副产品、工业产品专业批发市场。加快启动宜春中心城区现代商贸中心建设，做大做强樟树中药材交易市场，加快建设丰城水产品批发市场。大力引进大型现代物流企业，设立区域性物流公司、专业化加工配送中心和商品采购中心。充分利用高安货运优势，建设面向全省、服务全国的货运物流配载中心。

着力打造宜春旅游品牌。大力培育旅游经济产业，不断壮大旅游生产力。围绕“心圈廊”谋划和布局旅游业，突出开发绿色生态、温泉疗养、佛教禅宗和名人名地等旅游产品。加快温汤——明月山、三爪仑、天柱峰、阁皂山等重点景区景点建设，鼓励和引导民间资本开发旅游业。加大旅游宣传和市场促销力度，扩大宜春旅游影响。认真做好农运会旅游接待，启动中国优秀旅游城市创建工作。

（七）以人为本，提升素质，推动社会事业全面进步

积极实施科教兴国战略和可持续发展战略，加快大文化产业建设，推动经济和社会协调发展。

构筑科技和人才高地。加快科技创新步伐，加大科技投入，推动科研机构转制，完善科技进步激励机制，加大科技成果转化力度，大力发展科技中介服务和民营科技企业。加强知识产权保护和管理，普及科学知识，认真实施制造业信息化工程。鼓励企业建立自主创新的研发机构，促进产品更新和产业升级。落实引进高层次人才的优惠政策，逐步提高本市高层次人才的待遇，营造富集人才的城市形象和社会氛围。深化人事、分配制度改革，加强人才资源能力建设，完善对各类人才的选拔任用、考核评价、分配激励机制。

坚持优先发展教育。巩固提高义务教育，确保中小学教师工资、中小学生人均公用经费按时足额到位，确保以县为主的义务阶段教育管理体制改革全面到位。抓紧中小学危房改造，加快农村教育发展。加速发展高中段教育和职业教育，加速推行多元化的教育投资体制。加快市教育园区建设，做大做强宜春中学品牌。支持宜春职业技术学院、长江理工专修学院发展。加快宜春学院建设，多形式引进高层次人才，建设重点学科和重点实验室，尽快申报硕士点，开辟与国外大学联合办学的通道，提高办学质量，提升办学品质。抓紧组建教育后勤服务集团，实现教育系统后勤产业资源共享和优势互补。

推进各项社会事业发展。按照建设“文化大市”要求，推进文化创新，发展文化产业，繁荣文化事业。进一步加强社区文化建设和农村精神文明建设；积极整合文化资源，打造天文和禅宗文化品牌；鼓励外资、社会资本兴建、改造各类文化娱乐场所；开展文化市场专项整治，引导市场健康发展。大力开展全民健身活动，继续加大竞技体育后备人才培养力度，加快体育产业运作，充分利用现有体育设施，承接大型赛事和开展多种经营，提升体育产业的经济效益。加快公共卫生救治体系和疾病预防控制机构建设，加大农村卫生投入、卫生支农和卫生扶贫力度，积极稳妥地推进农村新型合作医疗改革。加快广电基础设施建设，加大网络整合力度，形成资源共享、事业互助的广播电视机制。整合全市报业和印刷业资源，组建报业集团，做大做强新闻传媒业。

大力实施可持续发展战略。深入推进人口与计划生育综合改革，完善目标责任制，加强基层基础工作，稳定低生育水平和现行生育政策，大力提高出生人口素质。合理开发和节约使用自然资源，健全资源的有偿使用制度，加大土地市场秩序整顿力度，依法保护耕地，从严控制城乡建设用地。加强生态环境保护和建设，坚持环境与发展统筹决策，大力开展植树造林，保护生态公益林，切实改善环境质量。以创建生态城市为目标，加大城市环境综合整治力度，严格综合执法，严格环保审批制度，继续清理整顿不法排污企业，从源头上控制新上污染企业和项目。

积极支持工会、共青团、妇联等群众团体开展工作，全面贯彻党和国家的民族、宗教、侨务政策，做好拥军优属和对台工作。加强国防教育、国防动员、民兵预备役和人防工作，关心和支持妇女、儿童、老龄、残疾人事业，做好统计、审计、档案、史志、气象和第一次经济普查工作。

（八）改进作风，依法行政，加强政府自身建设

按照勤政、务实、高效、廉洁的要求，进一步加强政府自身建设，不断提高管理经济和社会事务的能力和水平。

在转变作风上下功夫。坚决反对官僚主义和形式主义，敢于面对困难，善于破解难题，对看准了的事、定下来的事，一抓到底，抓出实效，确保政令畅通。坚持求真务实，多谋实干，善谋实干，努力提高政府工作效率，以工作的高效率来提高经济效率。坚持把人民群众的利益和要求，作为政府工作的出发点和落脚点，认真帮助解决群众生产生活中的实际问题。坚持解放思想，与时俱进，不断增强把握形势发展变化、抢抓发展机遇的能力，增强抓重点、抓落实、抓突破的能力。

在廉洁从政上下功夫。政府全体人员特别是领导干部，要时刻牢记“两个务必”，忠实履行“三个代表”重要思想，始终保持谦虚谨慎、不骄不躁的作风，保持艰苦奋斗的作风。加强政府机关工作人员的教育管理，自省自重，严于律己，塑造廉洁政府形象。加强监督检查，强化权力制约，切实纠正部门和行业不正之风，坚决杜绝“政府权力利益化，部门权力个人化”的现象。加快实施国库集中收付制度，扩大政府采购规模和范围，从源头上预防和解决腐败问题。严格落实党风廉政建设责任制，加大对违纪违法问题的查处力度，坚决清除腐败分子。

在依法行政上下功夫。自觉接受人大的法律监督和政协的民主监督，认真执行市人大议案决议，办理好人民代表的建议、批评、意见和政协委员的提案、建议案，认真听取各民主党派、工商联、无党派人士和人民团体的意见和建议，畅通社情民意渠道，尊重人民群众的知情权、参与权和监督权。完善法律服务体系，加强法律援助。继续强化行政执法监督检查，公正办理行政复议案件。

加强重点工程建设，对经济建设和社会发展形成有力支撑。着力实施“九大工程”，进一步夯实发展的基础。

1、能源建设工程。积极配合丰城电厂二期工程扩建，争取年内开工建设。做好靖安洪屏抽水蓄能电站的前期工作，争取国家早日立项。

2、重点工业项目工程。支持罗宾公司20万立方米中密度纤维板扩建工程，年底前基本结束设备安装调试。加快江西盐矿30万吨真空制盐项目建设，力争年底前竣工投产。抓紧江西同济丰宇建材公司20万立方米新型墙体材料一期工程建设。全面完成投资3亿元的江西恒泰塑业有限公司的建设。抓好丰城兰丰水泥、上高华夏水泥、高安红狮水泥等100万吨以上水泥项目建设。加快宜丰英田汽车有限公司10万辆汽车制造项目建设。

3、中心城建设工程。争取宜春中心城区污水处理厂一期工程开工建设，袁河水厂建成供水。全面完成宜春大道、北外环路和鼓楼路改造，完成迎宾公园、袁山公园、状元洲公园建设，启动新行政中心建设，力争主体框架工程在农运会召开前基本竣工。

4、国省道改造工程。加快宜万公路改造拓宽进度，确保8月底建成通车。抓紧320国道高安至万载、105国道丰城至樟树段改造的各项前期工作，争取早日动工建设。

5、电网改造工程。投资2.59亿元，开工建设以新建、改造13座110千伏输变电工程为主的县城电网改造。

6、水利设施除险加固工程。继续抓紧赣抚大堤、筠安堤等重点圩堤加固，做好樟树店下、高安樟树岭和丰城黄金等病险水库的除险加固。

7、学校建设工程。基本完成宜春中学高中部、宜春职业技术学院的校园建设，全面启动宜春学院三期工程建设。加快靖安中学、高安中学、丰城中学的新校区建设。

8、高科技研发工程。加快国家863计划项目“抗肝炎中药一类新药乌索酸及制剂的研究开发”、“优质高效杂交油菜新型授粉控制系统研究与杂交种选育”，国家“十五”重大科技专项“健心胶囊的开发与创新研究”，国家“火炬”计划“年产500吨果汁酒专用酵母JMJ2000”、“全自动免维护电力液压块式制动器”，国家高技术产业示范项目“南方油茶优良品种繁育及多梯度利用产业化”等项目的研发和成果转化。

9、无公害、绿色和有机农产品产业化工程。进一步加快无公害、绿色和有机农产品基地建设，做大一批龙头企业。

“群众利益无小事”。按照民本性、带动性和可操作性的原则，切实抓好便民、利民、惠民的“十大实事”，把实事办好，把好事办实。

1、增加就业岗位。实施积极的就业政策，加快劳动力市场建设，联贯城乡职业介绍网络。全市城镇新增从业人数2.2万人，解决再就业人数2.3万人，省外劳务输出55万人。

2、扶助弱势群体。解决困难群众生产生活问题，重点实施扶助贫困、扶助就业、扶助就学、扶助救灾、扶助病残的“五助”工程。继续开展干部扶贫帮困活动，做好城市居民最低生活保障，落实优惠政策；对困难企业军转干部设立医疗救助基金；发挥特困职工帮扶中心和再就业小额贷款担保中心的作用；拿出公益性岗位解决低保对象就业；多方筹措资金，使受灾灾民得到及时救助；开展帮扶结对活动，解决病残人员的生活困难。

3、改善环境质量。全面开展城市环境综合整治，着力抓好污水、油烟、粉尘、汽车尾气治理，严格控制居民集中区噪音污染，切实加强饮用水源保护。

4、改造乡村公路。全年完成乡村公路改造600公里，新增通油（水泥）路行政村300个以上。

5、建设疾病控制和医疗救治体系。上半年建成市级疾病预防控制中心、医疗急救治中心和县市区疾病预防控制中心，年底前建好市级传染病医院和县市传染病区。

6、改造农村中小学危房。全年投入6718万元，用于全市农村中小学18.3万平方米的危房改造。

7、切实解决农村人畜饮水问题。投资1570万元，解决6.3万农村人口的饮水困难。

8、加快农村卫生改厕步伐。建成1.5万座无害化厕所。

9、积极推广农村沼气。确保完成1万个建池任务。

10、完善中心城配套设施。加快城市防洪工程建设步伐，3月底前修筑好橡胶坝，抬高秀江水位。搞好垃圾中转站、医疗垃圾处理厂、公共厕所、停车场、农贸市场、化成岩公园、状元洲公园建设，彻底解决城区的积水问题。进一步加快城市主干道和小街小巷的改造和亮化，达到路通、水畅、灯亮。城区路灯亮化率达到98%。

完成上述目标和任务，要认真研究和把握经济发展的内在规律，在指导思想和实际工作中切实做到：

——正确认识赣西经济中心城市的定位。跳出行政区划上的中心概念意识，按照城市经济发展的内在规律和城市发展理论，主动将宜春融入赣西经济带中考虑，通过资源、信息、资金、人才的共享和互动，联合萍乡、新余建成江西最有发展潜力和生机活力的城市经济带。在产业结构上发挥生态优势，发展生物制药、电子信息等技术密集型和服装、玩具等劳动密集型产业，做强机械等对环境污染小的产业，进一步做大文化、教育、体育、医疗、商贸物流和旅游产业，将宜春建设成为赣西文化、商贸、旅游休闲中心。

——努力提高资金运作和资源整合水平。动用一切可能的资金和资源，精心操作，集中力量办大事。在六个环节上政府要有所作为。一是多渠道向外融资，适度负债经营。重点抓好国家开发银行贷款、省国投财产信托融资工作，加紧组建地方合作银行，在大财团和民众融资上求得新的突破，解决资金紧张的问题。二是站在全局高度整合各类资源，加快社会公用事业建设和社会公共产品的有效供给，改善投资环境。三是大力推进招商引资，重点发展工业园区、专业批发市场和大文化产业。四是通过工业园区、专业批发市场和大文化产业的带动，促进第三产业发展，提高城市经济实力，做旺城市人气。五是随着城市人口的增加，促进城市扩张，提升土地资源、城市资源的价值。六是在确保归还融资的基础上，增加新的投入。从而形成良性循环的资金链。

——统筹本级与县市区的发展。调整市政府工作重心，集中力量做大做强市本级，同时为县市区创造良好的发展环境。对本级而言，切实做到“履行职责，依法行政，统筹资源，取信于民”，履行职责，就是坚决贯彻执行中共中央、国务院、省委、省政府以及市委确定的路线、方针和政策；依法行政，就是政府必须按照法治的要求行使职权，做到政务公开；统筹资源，就是站在全局高度，整合各类有形无形资产和资源，加快推进工业化、城市化和农业产业化进程；取信于民，就是处处为民着想，事事为民谋利，做到决策前尊重民意，决策后雷厉执行，努力打造诚信政府。对县市区而言，切实做到“信任、放权、服务、指导”。凡是县市区政府自己能干好的事，就放手让县市区政府去干；凡是省里明确要放的权力，市政府一律下放，还权于基层和企业，对仍然有令不行、有禁不止的人和事，将坚决纠正和严肃处理。切实减少各种会议、文件、检查和一些不必要的调研活动，严格会议、检查的审批，凡涉及县市区政府领导参加的会议，一律报市政府审批，部门召开的会议也将严格控制。切实加强对县市区政府工作的指导，帮助县市区政府协调解决发展中的困难和问题。县市区政府必须按照市政府要求开展工作，以局部的发展支持全局的发展。

——正确处理发展与稳定的关系。在战略上坚持以发展为目的，以发展保持稳定；在实际操作中，把稳定放在首位，以稳定促进发展，打造“平安宜春”。切实抓好安全生产，健全安全生产机构，加强安监队伍建设，深化安全生产专项整治，建立安全生产长效管理机制，确保实现“两杜绝一减少”（杜绝特大死亡事故，杜绝校园内死亡事故，减少重大死亡事故和一般死亡事故）。认真贯彻落实减轻农民负担政策，切实开展涉农收费专项治理，凡因农民负担问题引发严重事件和恶性案件的，对有关责任人要严肃查处。加大严打整治斗争力度，大力抓好社会治安综合治理，认真做好信访和纠纷调处工作，妥善处理人民内部矛盾。确保全国农运会举办期间不发生重特大事故和恶性案件。

各位代表，回首宜春的发展历程，我们豪情满怀；展望宜春的未来，我们深感责任重大。朝着全面建设小康宜春这个光辉的目标，我们已经迈出了坚实的步伐，必将迈出更加自信的步伐。让我们紧密团结在以胡锦涛同志为总书记的党中央周围，在中共宜春市委的坚强领导下，以奋发有为的精神状态、求真务实的工作作风，创造新的发展业绩，开创新的发展局面，为全面建设小康宜春而努力奋斗!
